# A novel cutoff for the waist-to-height ratio predicting metabolic syndrome in young American adults

**DOI:** 10.1186/s12889-016-2964-6

**Published:** 2016-04-01

**Authors:** Adam D. Bohr, Kelly Laurson, Matthew B. McQueen

**Affiliations:** University of Colorado Boulder, 4185 47th St., Unit C, Boulder, CO 80301 USA; Illinois State University, 347 S. University Street, McCormick 151B, Normal, IL 61761 USA; Department of Integrative Physiology, University of Colorado Boulder, 354 UCB, Clare Small 102, Boulder, CO 80309-0354 USA

**Keywords:** Waist-to-height ratio, Cutoff, Obesity, Epidemiology, Metabolic syndrome

## Abstract

**Background:**

Recent studies have shown the enhanced diagnostic capability of the waist-to-height ratio (WHtR) over BMI. However, while a structured cutoff hierarchy has been established for BMI, a rigorous analysis to define individuals as obese using the WHtR has not been performed on a sample of American adults. This study attempts to establish a cutoff for the WHtR using metabolic syndrome as the outcome.

**Methods:**

The study sample consisted of individuals that were part of the National Longitudinal Study of Adolescent Health (Add Health). The final sample for analysis consisted of 7 935 participants (3 469 males, 4 466 females) that were complete respondents for the variables of interest at Wave IV. The participants ranged from 24.55-33.60 years. Weighted and unweighted receiver operator characteristics (ROC) analyses were performed predicting metabolic syndrome from the WHtR. Cutoffs were chosen using the Youden index. The derived cutoffs were validated by logistic regression analysis on the Add Health participants and an external sample of 1 236 participants from the National Health and Nutrition Examination Survey (NHANES).

**Results:**

The ROC analysis resulted in a WHtR cutoff of 0.578 (Youden Index = 0.50) for the full sample of complete respondents, 0.578 (Youden Index = 0.55) for males only, and 0.580 (Youden Index = 0.50) for females only. The area under the curve was 0.798 (95 % CI (0.788, 0.809)) for the full sample of complete respondents, 0.833 (95 % CI (0.818, 0.848)) for males only, and 0.804 (95 % CI (0.791, 0.818)) for females only. Participants in the validation sample with a WHtR greater than the derived cutoff were more likely (Odds Ratio = 9.8, 95 % CI (6.2, 15.3)) to have metabolic syndrome than those that were not.

**Conclusion:**

A WHtR cutoff of 0.580 is optimal for discriminating individuals with metabolic syndrome in two nationally representative samples of young adults. This cutoff is an improvement over a previously recommended cutoff of 0.5 as well as other cutoffs derived from international samples.

**Electronic supplementary material:**

The online version of this article (doi:10.1186/s12889-016-2964-6) contains supplementary material, which is available to authorized users.

## Background

Obesity is an increasing worldwide problem and known risk factor for the development of several chronic diseases [1, 2]. BMI has traditionally been used as an indicator of weight status and cardiometabolic risks associated with being overweight. However, despite being widely utilized as the default for determining weight status, evidence suggests an inconsistent ability of the BMI to predict disease risk. Several studies have shown individuals that were overweight or obese based off of BMI cutoffs were actually at reduced mortality compared with normal weight individuals, while only those that were severely obese or underweight were at an increased risk [3–5].

For these reasons, alternative measures of weight status that take the distribution of body mass into consideration could be superior diagnostic measures for identifying cardiovascular and metabolic disease risk. One of these measures is the waist-to-height ratio (WHtR), which has been shown to outperform BMI and waist circumference (WC) in discriminating risk of hypertension, diabetes, and cardiovascular disease risk [6, 7].

Given the diagnostic potential of the WHtR, it is important that an appropriate cutoff is established that can identify an individual as overweight or at risk for cardiovascular disease outcomes. An international cutoff of 0.5 has been proposed in the past [6, 7]. In addition, cutoffs have been established in Asian populations. For example, a study of Beijing adults established WHtR cutoffs of 0.51-0.53 and 0.48-0.50 in men and women, respectively [8]. Another study conducted in China suggested cutoffs for severe obesity of 0.54 in men and 0.57 in women [9]. However, it has been shown that WHtR may discriminate differently in Asian versus non-Asian populations, and rigorous analysis has not been performed to establish a cutoff that can be applied to obesity research for populations in the United States [10].

The primary objective of this study was to establish such a cutoff using a sample of Wave IV participants from National Longitudinal Study of Adolescent Health (Add Health). In addition, we tested the validity of the derived cutoff using another sample of young adults from the National Health and Nutrition Examination Survey (NHANES). The results of this study could be used in future Add Health studies to aid in analyses when the WHtR is the outcome variable. In addition, it could be used as a metric in other studies using young adults in the United States when obesity or risk of obesity is an outcome of interest.

## Methods

### Participants

The study utilized a sample of individuals from Add Health [11]. Add Health is a longitudinal study that investigates how social and environmental factors may influence health and has followed a cohort of individuals through four waves of interviewing and testing since its inception during the 1994-1995 school year. The current study utilized variables from Wave IV testing. The full Add Health study consisted of 20 792 participants. Add Health participants provided written informed consent for participation in all aspects of Add Health in accordance with the University of North Carolina School of Public Health Institutional Review Board guidelines.

The initial subsample of this population consisted of individuals that were part of blood glucose homeostasis measurements (n = 15 701). Additionally, the packages used for analyses in the current study required complete information for all variables used, and a substantial amount of the respondents had missing data for some of the Wave IV measurements and variables. Any respondent that did not have complete information for all of the variables used was not part of the final analysis. The final sample for analyses consisted of 7 935 participants. For purpose of comparison, descriptive statistics are presented for both this sample as well as the respondents that were removed in Table 1.

**Table 1:** Descriptive Statistics for Participants from the Add Health Study, 1994-2008

Variable	Complete Cases (n = 7 935)		Incomplete Cases (n = 7 766)		*P Value*
	Count	%	Count	%	
Sex					
Males	3 469	43.7 %	3 883	50.0 %	<0.0001
Females	4 466	56.3 %	3 881	50.0 %	<0.0001
Missing/Other	0	0.0 %	2	0.0 %	
Race					
NonHispanic Caucasian	4 624	58.3 %	4 006	51.8 %	<0.0001
NonHispanic African American	1 530	19.3 %	1 928	24.9 %	<0.0001
NonHispanic Asian/Native American	584	7.4 %	498	6.4 %	0.023
Hispanic	1 197	15.1 %	1 301	16.8 %	0.003
Smoking					
Smokers	2 833	35.7 %	2 724	35.7 %	0.997
Metabolic Outcomes					
High Waist Circumference	4 206	53.0 %	3 768	49.5 %	<0.0001
High Triglycerides	2 779	35.0 %	1 958	34.6 %	0.600
High Blood Glucose	2 647	33.4 %	2 251	34.8 %	0.067
Low High Densitiy Lipoprotein	1 991	25.1 %	1 421	24.8 %	0.651
Hypertension	1 553	19.6 %	1 515	20.4 %	0.225
Metabolic Syndrome	1 853	23.4 %	1 262	23.8 %	0.534
	Mean	SE	Mean	SE	*P Value*
Age	28.65	0.018	29.4	0.021	<0.0001
Waist-to-Height Ratio	0.5796	0.001	0.577	0.001	0.121
Body Mass Index	29.15	0.084	29.13	0.088	0.165
SE (Standard Error)					

### Race, sex, age, and smoking variables

Racial classification used the variable “AH_RACE” and combined the groups “nonHispanic Asian” and “nonHispanic Native American” due to sparsely populated cells for those groups. Race was treated as a factor, comparing “nonHispanic African Americans”, “nonHispanic Asian/Native American”, and “Hispanic” to the referent category, “nonHispanic Caucasian.” Age was measured continuously in years. Average age of the participants at Wave IV was 28.65 (SD = 1.60) years and ranged from 24.55-33.60 years. Smoking was discrete and measured in the amount of days smoked in the last month [11]. Sex was dichotomous with males coded as “1” and females coded as “2.” The names of the source variables used can be found in Additional file 1: Table S1.

### Cardiometabolic outcomes

For description of the collection of cardiometabolic measurements in Add Health, please refer to the supplementary information to this manuscript (Additional file 2: Supplementary Information).

When possible, the National Cholesterol Education Adult Treatment Panel III (NCEP/ATP III) diagnostic criteria for metabolic syndrome were used for classification purposes [12, 13]. Individuals are considered to have metabolic syndrome if three or more of five risk factors are present. These include high waist circumference, high blood pressure, high fasting blood glucose, high triglyceride (TG), and low HDL [12, 13]. The NCEP/ATP III criteria were applied for determination of both high waist circumference (men: > 102 cm, women: > 88 cm) and blood pressure (≥135/≥85 mm HG). However, due to the issues related to fasting time for blood glucose measurements and the fact that only deciles were released for lipid measurements, other constructs for risk in these areas were used.

In place of fasting blood glucose, glycolated hemoglobin (HbA_1C_) was used as a measure of glycemic homeostasis. Individuals with HbA_1C_ > 5.7 % were classified as pre-diabetic or diabetic, which roughly corresponds to fasting blood glucose of ≥ 100 mg/dl [14]. Because no absolute measures for TG or HDL were released, decile ranks were used to classify individuals for these two risk factors. Previous research from 2003-2006 reported a prevalence of hypertriglyceridemia of 29.6 % and 17.8 % in 20-39 year old males and females, respectively. The same study reported a prevalence of low HDL of 21.4 % and 29.4 % in 20-39 year old males and females, respectively [14]. Therefore, males that were in the top three deciles and females that were in the top two deciles for TG were classified has having this risk factor. Males that were in the bottom two deciles and females that were in the bottom three deciles for HDL were classified as having this risk factor.

Individuals that had three or more of the risk factors were classified as having metabolic syndrome, resulting in an overall prevalence of 23.35 % of individuals that were part of the final cutoff analysis. The sex specific prevalence was 28.16 % for males and 19.61 % for females. These are slightly higher than those reported in Ervin et al. (20.3 % in males aged 20-39 years, 15.6 % in females aged 20-39 years) [14].

Height (cm) and waist circumference (cm) were extracted from the Add Health Wave 4 anthropometric measurements. Waist circumference was measured to the nearest 0.5 cm at the superior border of the iliac crest. The WHtR metric was calculated by dividing waist circumference by height.

### ROC analysis: unweighted

Due to limitations of the statistical software package used to identify cutoffs, we initially conducted unweighted ROC analysis which did not account for sampling weights, strata, or clusters. Analysis using metabolic syndrome as the classification variable and WHtR as the continuous predictor variable was performed to determine the appropriate WHtR cutoff. Both sex specific and full sample analyses were performed. The optimal cutoffs in this study were determined by the Youden Index, which is defined as Sensitivity + Specificity - 1 [15]. All analyses and calculations were done using R version 2.15.3 via the RStudio platform, version 0.97.320 and used the R package “OptimalCutpoints” [16, 17].

### ROC analysis: weighted

We also conducted weighted ROC analysis, which accounted for sampling weights, strata, and clustering. Add Health employs a multi-stage, stratified, and clustered sampling strategy. In addition, certain racial, ethnic, and socioeconomic groups were intentionally over-sampled [18]. In order to account for the weights, strata, and clusters, the R package “Survey” was used to create a study design value that could be applied to different types of analysis [19].

To conduct the weighted ROC analysis, “Survey” was used to generate weighted contingency tables that account for weights, strata, and clustering variables. Step 1 involved creating potential cutoffs for the WHtR from 0.5-0.6 at a resolution of every hundredth. Step 2 involved generating contingency tables for these cutoffs and presence of metabolic syndrome, and step 3 involved calculating the sensitivity and specificity of each potential cutoff. For both the full and male only samples, the Youden Index increased until 0.57 and then began to decrease. Cutoffs were then created from 0.56 to 0.58 at a resolution of every thousandth, and steps 2 and 3 of the analysis were repeated. For females, the Youden Index increased until 0.59 and then decreased. Cutoffs were created ranging from 0.575 to 0.595 at a resolution of every thousandth, and steps 2 and 3 of the analysis were repeated.

“Survey” was also used to calculate the area under the curve (AUC) for the optimal cutoffs by generating logistic regression models to predict metabolic syndrome from the derived cutoffs. Concordance was then calculated from these models to determine the AUC.

### Logistic regression model

Separate analysis was performed to test the derived cutoffs while controlling for confounding factors. Indicator variables were created for whether or not an individual was above the WHtR cutoff identified in the ROC analysis. Weighted and unweighted logistic regression models were constructed for the full sample, males only, and females only. These models predicted presence of metabolic syndrome from the WHtR cutoff indicator, race, sex, age, and smoking.

### External validation

Finally, the derived cutoffs were tested using an external sample from NHANES for the 2005-2006 and 2007-2008 collection periods. Analysis was restricted to complete respondents for the variables of interest and to individuals aged 25-35 years for comparison with the Add Health participants. This resulted in a sample of 1 236 respondents. A construct for metabolic syndrome was created, and the following contingency tables were generated predicting metabolic syndrome from the derived cutoffs: Unweighted, full sample, WHtR > 0.580; Unweighted, males only, WHtR > 0.578; Unweighted, females only, WHtR > 0.580; Weighted, full sample, WHtR > 0.580. Finally, logistic regression analysis was used to test the cutoff’s ability to predict metabolic syndrome while controlling for race, age, sex, and smoking status. A complete description of the variables (Additional file 3) used as well as descriptive statistics (Additional file 4) for this sample can be found in the supplementary information to this manuscript.

## Results

Descriptive statistics for the sample of complete respondents as well as the respondents that were removed can be found in Table 1. *P* values from independent tests of proportions and means are displayed to test for systematic differences between the complete respondents and the incomplete respondents. The two samples differed in the demographic categories, with the most pronounced differences between males and females (*P* < 0.0001), “nonHispanic Caucasian” and “nonHispanic African American” racial categories (*P* < 0.0001), and age (*P* < 0.0001). However, they did not differ on smoking status (*P* = 0.997) and all but one of the metabolic outcomes (Waist Circumference, P < 0.0001). Prevalence of metabolic syndrome was not significantly different (*P* = 0.534) between complete respondents and incomplete respondents. Finally, the samples did not differ in measures of weight status or weight distribution (BMI: *P* = 0.165, WHtR *P* = 0.121).

**Fig. 1:**
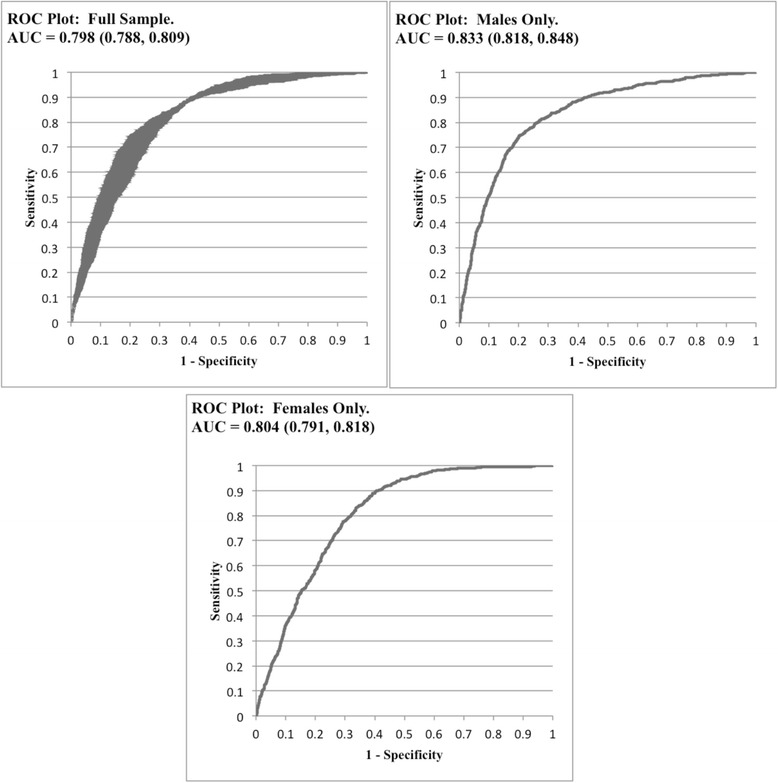
ROC (Receiver Operator Characteristics). AUC (Area Under the Curve)

The results of the unweighted ROC analysis are presented in Table 2. In addition to sensitivity and specificity, point estimates and 95 % confidence intervals for positive predictive value (PPV), negative predictive value (NPV), positive likelihood ratio (PLR), negative likelihood ratio (NLR), and odds ratios (OR) were calculated. PPV is the probability that a person that tests positive for the disease outcome is an actual case, while NPV is the probability that a person that tests negative for the disease outcome is not a case. PLR and NLR are measures of how much a test result will change the odds of having a disease or not having a disease. The ROC plots for this analysis are presented in Fig. 1.

**Table 2:** Unweighted ROC Analysis Predicting Metabolic Syndrome from Waist-to-Height Ratio: Add health Study 1994-2008

Measure	Full Sample (n = 7 935)	Males Only (n = 3 469)	Females Only (n = 4 466)
Cutoff	0.578	0.578	0.580
Sensitivity	0.82 (0.79, 0.84)	0.75 (0.72, 0.77)	0.89 (0.87, 0.91)
Specificity	0.68 (0.67, 0.70)	0.80 (0.78, 0.81)	0.61 (0.59, 0.62)
PPV	0.44 (0.42, 0.48)	0.59 (0.57, 0.63)	0.36 (0.34, 0.41)
NPV	0.92 (0.91, 0.93)	0.89 (0.87, 0.90)	0.96 (0.95, 0.96)
PLR	2.56 (2.41, 2.71)	3.69 (3.39, 4.03)	2.26 (2.15, 2.36)
NLR	0.27 (0.24, 0.31)	0.32 (0.28, 0.35)	0.18 (0.15, 0.22)
Odds Ratio	9.4 (8.2, 10.7)	11.4 (9.6, 13.6)	12.3 (9.9, 15.4)

(95 % Confidence Interval) PPV (Positive Predictive Value) NPV (Negative Predictive Value) PLR (Positive Likelihood Ratio) NLR (Negative Likelihood Ratio)

The ROC analysis resulted in a WHtR cutoff of 0.578 (Youden Index = 0.50) for the full sample of complete respondents, 0.578 (Youden Index = 0.55) for males only, and 0.580 (Youden Index = 0.50) for females only. The AUC was 0.798 (95 % CI (0.788, 0.809)) for the full sample of complete respondents, 0.833 (95 % CI (0.818, 0.848)) for males only, and 0.804 (95 % CI (0.791, 0.818)) for females only. Individuals that were above the cutoff of 0.578 were 9.4 (95 % CI (8.22, 10.65)) times more likely to have metabolic syndrome. Restricting to males only, those that were above the cutoff of 0.578 were 11.4 (95 % CI (9.61, 13.60)) times more likely to have metabolic syndrome. Finally, females that were above a WHtR of 0.580 were 12.3 (95 % CI (9.88, 15.38)) times more likely to have metabolic syndrome.

The results of the weighted ROC analysis are presented in Table 3. This analysis once again resulted in a cutoff of 0.578 (Youden Index = 0.51) in the full sample while the cutoff for males only was 0.576 (Youden Index = 0.58). The cutoff for females only increased to 0.594 (Youden Index = 0.51), though, there was a very narrow range of values for the Youden Index between the established cutoff of 0.594 and the cutoff from the initial analysis 0.580 (Youden Index = 0.50). In addition, specificity in the female sample was the only diagnostic measure that differed significantly between the unweighted (0.61, 95 % CI (0.59, 0.62)) and weighted analysis (0.65, 95 % CI (0.63, 0.66)). However, if the cutoff of 0.580 had been used, the specificity (0.60, 95 % CI (0.57, 0.62)) would not have differed from the unweighted analysis. The AUC was 0.794 for the full sample, 0.817 for males only, and 0.805 for females only.

**Table 3:** Weighted ROC Analysis Predicting Metabolic Syndrome from Waist-to-Height Ratio: Add health Study 1994-2008

Measure	Full Sample (n = 7 935)	Males Only (n = 3 469)	Females Only (n = 4 466)
Cutoff	0.578	0.576	0.594
Sensitivity	0.82 (0.81, 0.84)	0.78 (0.75, 0.80)	0.86 (0.83, 0.88)
Specificity	0.69 (0.68, 0.70)	0.80 (0.78, 0.81)	0.65 (0.63, 0.66)
PPV	0.44 (0.42, 0.46)	0.59 (0.56, 0.61)	0.36 (0.34, 0.38)
NPV	0.93 (0.92, 0.94)	0.91 (0.89, 0.92)	0.95 (0.94, 0.96)
PLR	2.64 (2.53, 2.75)	3.79 (3.48, 4.12)	2.42 (2.30, 2.55)
NLR	0.26 (0.23, 0.28)	0.28 (0.25, 0.32)	0.22 (0.19, 0.26)
Odds Ratio	10.3 (8.5, 12.5)	13.6 (10.4, 17.7)	10.0 (8.6, 13.9)

(95 % Confidence Interval) PPV (Positive Predictive Value) NPV (Negative Predictive Value) PLR (Positive Likelihood Ratio) NLR (Negative Likelihood Ratio)

In separate analysis, logistic regression models were constructed to test the established cutoffs while controlling for confounding factors (Table 4). Each WHtR cutoff was a highly significant predictor (*P* < 0.0001) with the inclusion of constructs for race, smoking, age, and sex.

**Table 4:** Logistic Regression Predicting Presence of Metabolic Syndrome from WHtR Cutoff: Add Health Study, 1994-2008, n = 7 935

Unweighted	Full Sample		Females Only		Males Only	
	OR	*P Value*	OR	*P Value*	OR	*P Value*
WHtR Cutoff	12.0 (10.41, 13.74)	<0.0001	12.0 (9.64, 15.05)	<0.0001	11.7 (9.79, 13.96)	<0.0001
NonHispanic Caucasian	Ref	Ref	Ref	Ref	Ref	Ref
NonHispanic African American	1.3 (1.12, 1.57)	0.001	1.4 (1.14, 1.70)	0.001	1.2 (0.93, 1.52)	0.164
NonHispanic Native American/Asian	1.7 (1.36, 2.14)	<0.0001	1.8 (1.28, 2.50)	0.001	1.7 (1.23, 2.30)	0.001
Hispanic	1.2 (1.03, 1.44)	0.020	1.4 (1.11, 1.76)	0.004	1.1 (0.83, 1.34)	0.674
Smoking	1.0 (1.00, 1.01)	0.001	1.0 (1.00, 1.02)	0.003	1.0 (0.99, 1.01)	0.096
Age	1.1 (1.03, 1.11)	<0.001	1.1 (1.00, 1.11)	0.048	1.1 (1.03, 1.15)	0.002
Sex	0.4 (0.33, 0.42)	<0.0001	-	-	-	-
Weighted	Full Sample		Females Only		Males Only	
	OR	P Value	OR	P Value	OR	P Value
WHtR Cutoff	13.3 ( 10.79, 16.30)	<0.0001	10.6 (8.35, 13.44)	<0.0001	13.8 (10.58, 18.08)	<0.0001
NonHispanic Caucasian	Ref	Ref	Ref	Ref	Ref	Ref
NonHispanic African American	1.3 ( 1.14, 1.62)	0.001	1.5 (1.17, 1.95)	0.002	1.3 (0.88, 1.82)	0.213
NonHispanic Native American/Asian	1.4 ( 0.80, 2.30)	0.259	1.7 (1.03, 2.86)	0.040	1.2 (0.59, 2.26)	0.667
Hispanic	1.1 (0.86, 1.36)	0.505	1.5 (1.17, 1.98)	0.002	0.8 (0.59, 1.14)	0.230
Smoking	1.0 (1.00, 1.01)	0.052	1.0 (1.00, 1.02)	0.039	1.0 (0.99, 1.01)	0.393
Age	1.1 (1.01, 1.11)	0.023	1.0 (0.98, 1.11)	0.160	1.1 (0.99, 1.15)	0.059
Sex	0.4 (0.30, 0.42)	<0.0001	-	-	-	-

(95 % Confidence Intervals) OR (Odds Ratio)

### Validation results

The full results of the validation analyses are presented in the supplementary information (Additional file 5: Table S4 and Additional file 6: Table S5) to this manuscript. The overall cutoff of 0.580 resulted in a Youden Index of 0.49 in the unweighted analysis and 0.52 in the weighted analysis. In addition, the derived cutoffs were highly significant predictors of metabolic syndrome in the weighted and unweighted full sample logistic regression models as well as the unweighted sex-specific logistic regression models.

## Discussion

Recent research has established WHtR as a more useful diagnostic measure for overweight than BMI. This could be due in part to the fact that BMI does not take lean mass or the distribution of body weight into consideration. Fat distribution is known to factor greatly into risk of cardiovascular disease and early death, as visceral fat is more metabolically active and associated with greater systemic inflammation when compared with subcutaneous adipose tissue [20, 21]. As such, visceral adiposity is more causally related to insulin resistance, hypertension, and dyslipidemia [22, 23].

Add Health is an ideal sample and study design to conduct cutoff research and has several features that enhance the validity as well as the utility of our findings. First, WHtR, as well as all of the metabolic outcomes, were measured in the field by trained technicians rather than self-reported. In addition, the design of the Add Health study allows for opportunity to make true population inferences as it is a nationally representative study with variables in place to account for complex sampling strategy. Finally, Add Health is a longitudinal study with several waves of data collection and includes a vast array of associational parameters for body composition and weight distribution. The richness of this data may allow researchers to observe how the cutoff may change as the sample ages in addition to how behavioral, genetic, and sociodemographic factors may impact risk of being overweight as defined by WHtR.

We opted to use metabolic syndrome as our disease outcome as it incorporates a host of cardiovascular outcomes that are linked to mortality. The prevalence of metabolic syndrome that we observed in men (28.2 %) and women (19.6 %) was slightly higher than had been reported in previous studies [14, 24]. Ervin et al. (2009) reported a prevalence of 20.3 % and 15.6 % in 20-39 year old males and females, respectively, while Ford et al. (2004) reported 16.5 % and 19.1 % in 20-39 year old males and females, respectively. Waist circumference was by far the largest source of the difference observed, as our sample had a prevalence of abdominal obesity of 37.1 % and 65.3 % in males and females, respectively. Both of these were significantly higher than reported by Ervin et al. (32 % and 49.8 % in males and females, respectively).

Another element of our sample worth commenting on is whether or not there were significant differences between the individuals that were complete respondents for the variables we were observing and those that were not included in our analysis. It is possible that selection bias could have occurred if there were systematic differences between individuals that were complete respondents, which would make our results less generalizable. The individuals that were selected had significantly higher occurrence of high waist adiposity. In addition, there were racial and sex differences between the groups, with the final sample having fewer males and “nonHispanic African Americans” and more “nonHispanic Caucasians” than the sample of participants that were not selected. However, none of the other metabolic outcomes differed between the two samples and there were no differences between WHtR and BMI of the participants. In addition, our decision to conduct sex specific and full sample analyses reduces the chance that differences in proportion of males and females could affect the final analyses. Finally, we tested the cutoffs established while controlling for race, and these cutoffs stayed highly significant in all analysis.

Previous research had suggested a potential international cutoff of 0.5. However, investigation of the Add Health sample indicates that this may not be appropriate. This cutoff is significantly below both the median (0.559) and mean (0.578, SE = 0.001) WHtR of the sample and would classify the vast majority of the sample as overweight. If used to predict metabolic syndrome, this cutoff performs relatively poorly (Sensitivity: 0.97, 95 % CI (0.96, 0.98); Specificity: 0.29 95 % CI (0.28, 0.30)) compared to the cutoffs we’ve established.

We performed both analysis that accounted for sampling strategy (ie. weights, strata, and cluster) and analysis that did not. There were slight differences in the resulting cutoffs between the weighted analysis and the unweighted analysis, though none of the diagnostic measurements differed between the two sets of analysis. The weighted analysis resulted in a cutoff of 0.576 for males and 0.594 for females while the unweighted analysis resulted in cutoffs of 0.578 and 0.580 for males and females. The difference in cutoffs for females was somewhat concerning. However, there was a very narrow range of the Youden Index (0.50-0.51) for cutoffs from 0.580 to 0.594 in the weighted analysis, which would indicate that cutoffs in this range would have roughly the same diagnostic capability.

Given that two different sets of analyses were performed, and that sex-specific cutoffs have been proposed in the past, we feel that it is appropriate to recommend a range of WHtR cutoffs for males (0.575-0.580) and females (0.580-0.595) to classify individuals as overweight. However, considering that there were only slight differences between the results of the weighted and unweighted analysis, as well as the similar diagnostic capabilities within a narrow range of cutoffs, we also feel that it is appropriate to additionally report a single, overall cutoff of 0.58.

The results of the logistic regression support the use of these cutoffs and also show their physiologic significance. The cutoffs remained highly significant predictors in all analyses even with the inclusion of race, sex, smoking status, and age. Additionally, the odds of metabolic syndrome increased drastically if an individual was above the cutoff. Males above the cutoff of 0.578 were greater than eleven times more likely to have metabolic syndrome than males that were not, while females that were above the cutoff of 0.580 were greater than twelve times more likely to have metabolic syndrome than females that were not. These results were similar in the weighted and unweighted analysis.

### Limitations

The primary limitation of the current study was the lack of absolute measures for HDL and TG. This prevented us from applying the NCEP/ATP III criteria for classification of metabolic syndrome. Several other studies that use metabolic syndrome apply the NCEP/ATP III criteria, so our inability to do so impairs the comparability of our results with other studies.

We were also limited in how we classified resondents as having high blood glucose. The lack of control over the fasting time of the participants made the results of the blood glucose less reliable. We were, however, able to use HbA_1C_ as an indicator of glucose status. While HbA_1C_ is the gold standard for glycemic homeostasis, it too deviates from the ATP III criteria and therefore limits our comparability with other studies.

In addition, while the logistic regression models included controls for race, sex, age, and smoking; they did not include other common confounding factors such as physical activity or socioeconomic status of the participants. As the primary purpose of these models was validation of the cutoffs and not variability explanation in metabolic syndrome, we felt it was appropriate to be parsimonious in covariate inclusion. Finally, we feel that the strength of the association, both statistically and physiologically, between the WHtR and metabolic syndrome is such that it is unlikely additional covariates would completely diminish the relationship.

The paper is limited by the cross-sectional nature of the data and study design. While Add Health itself is a longitudinal study, we utilized only data from Wave IV. This prevents us from drawing a causal relationship between the WHtR cutoff and metabolic syndrome.

Finally, a significant amount of the sample (7 776 respondents) was lost due to missingness of any one of the measures of interest. In addition, the final sample differed from removed respondents in racial and gender make-up. This impairs the ability of our findings to generalize to this particular population and introduces the potential for sampling bias. However, we feel that our use of sampling weights, strata, and cluster variables in analysis attenuates some of these issues. Finally, it is our contention that though there is likely some degree of sampling bias present, due to the fact that there were no differences between complete and incomplete respondents for the primary variables of interest (WHtR and metabolic syndrome) it is unlikely that the results are unreliable.

## Conclusion

Based on both weighted and unweighted analyses, we recommend a WHtR cutoff of 0.575-0.580 for males and 0.580-0.595 for females in addition to a single, overall cutoff of 0.58. These cutoffs can be used in prospective Add Health studies that use the WHtR as a predictor or outcome and provide a measure of the physiologic significance of differences in the WHtR. As the prevalence of metabolic syndrome has been shown to increase with age, future studies are warranted to determine age appropriate cutoffs. In addition, a more hierarchical cutoff structure similar to that of BMI could be developed. Our cutoff identified individuals that had metabolic syndrome, but cutoffs could be developed to identify those at low risk, moderate risk, and high risk. Finally, the Add Health study provides a unique opportunity to follow the cohort prospectively, which will allow us to observe the disease histories of individuals that are above or below these cutoffs.

## Data availability statement

All data used in this study is publicly available at the Add Health website: www.cpc.unc.edu/projects/addhealth.

### Supplementary information

Supplementary information is available at *BMC Public Health’s* website.

## Additional files

Additional file 1: Table S1. Add Health Source Variables Used for Analysis (XLSX 38 kb) Additional file 2: Supplementary information (DOCX 109 kb) Additional file 3: Table S2. NHANES Source Variables Used for Validation Analysis (XLSX 46 kb) Additional file 4: Table S3. Descriptives Statistics: Subsample of National Health and Nutrition Examination Survey, 2005-2008, n = 1 236 (XLSX 33 kb) Additional file 5: Table S4. Unweighted Logistic Regression Predicting Presence of Metabolic Syndrome from WHtR Cutoff: National Health and Nutrition Examination Survey, 2005-2008, n = 1 236 (XLSX 48 kb) Additional file 6: Table S5. Weighted Logistic Regression Predicting Presence of Metabolic Syndrome from WHtR Cutoff: National Health and Nutrition Examination Survey, 2005-2008, n = 1 236 (XLSX 44 kb)

## Abbreviations

BMI: Body mass index
WHtR: Waist-to-height ratio
WC: Waist circumference
ROC: Receiver operator characteristic
NHANES: National Health and Nutrition Examination Survey
NCEP/ATP III: National Cholesterol Education Adult Treatment Panel III
TG: Triglyceride
HbA_1C_: Glycolated hemoglobin
AUC: Area under the curve
PPV: Positive predictive value
NPV: Negative predictive value
PLR: Positive likelihood ratio
NLR: Negative likelihood ratio
OR: Odds ratio
