# Supplementary material for: A novel cutoff for the waist-to-height ratio predicting metabolic syndrome in young American adults
Source: BMC Public Health. 2016 Apr 1;16:295. doi: 10.1186/s12889-016-2964-6 (PMC4818465; doi:10.1186/s12889-016-2964-6)
Supplement: Additional file 2: — Supplementary information (DOCX 109 kb) [file 12889_2016_2964_MOESM2_ESM.docx]

SUPPLEMENTARY INFORMATION

*Cardiometabolic Outcomes Data Collection*

All cardiometabolic and anthropometric outcomes were measured by trained field interviewers as part of the Add Health study. Waist circumference was measured to the nearest 0.5 cm using a SECA 200 metric-increment circumference tape. Height was measured to the nearest 0.5 cm using a carpenter’s square and steel tape measurer. Weight was measured to the nearest 0.1 kg with participants fully clothed using a Health-o-meter 844KL High Capacity Digital Bathroom Scale. WHtR was derived by dividing a participant’s waist in cm by his or her height in cm.^1^

Systolic and diastolic blood pressures were measured three times on the right arm at 30-second intervals using a Microlife BP3MC1-PC-IB oscillometric blood pressure monitor and large adult cuff. The average of the 2^nd^ and 3^rd^ reading were recorded as the participant’s blood pressure. ^1^

Whole capillary blood for fasting blood glucose measurements was collected from participants via finger prick by trained field interviewers. However, the fasting times for these samples were not controlled, and fast time variability contributed significantly to determining whether or not a participant would be classified as pre-diabetic or diabetic (56.49% of individuals that fasted for at least eight hours, 5.54% of individuals that did not fast for at least eight hours). The glucose homeostasis measurements also included a measure of HbA_1C_^,^ which is considered the gold standard for glycemic homeostasis.^2^ In addition, this measure also classifies individuals as pre-diabetic or diabetic regardless of fast time.^3^ As such, this measure was used to classify participants as pre-diabetic or diabetic for determination of metabolic syndrome.

Whole capillary blood for triglycerides (TG) and high-density lipoprotein (HDL) measurements was collected via finger prick by trained field interviewers. Both TG and HDL were determined by cholesterol colorimetric assays. Absolute measurements were for neither TG nor HDL were released due to a variety of reasons as outlined in Add Health Wave IV Documentation: Lipids.^4^ Instead, participants were assigned a decile rank for these lipid measurements.

For more information on all of the cardiometabolic measurements used in this study, please refer to the online Add Health Wave IV Documentation: Cardiovascular and Anthropometric Measures, Glucose Homeostasis, and Lipids.^1-2, 4^

*NHANES Variables Used in Validation*

Sources variables used and descriptions can be found in Table S2. All instances of missingness or refusal to answer were removed from the data. The NCEP/ATP III were used to assign risk factors for high waist circumference (men: >102 cm, women >88 cm), high triglyceride (≥150 mg/dL), low HDL (men: <40 mg/dL, women, <50 mg/dL), and high blood pressure (≥135/≥85 mm Hg). However, to stay consistent with the initial analysis, the risk factor for high glucose was assigned if HbA_1C_ > 5.7 %. Three measurements were taken for systolic and diastolic blood pressure, and an average of those three measures was used to assign risk of high blood pressure. Metabolic syndrome was defined as the presence of three or more of the risk factors.

Race was defined as a factor in the logistic regression analysis, comparing “Mexican American”, “Other Hispanic”, “nonHispanic Black”, and “Other Race” to the referent group “nonHispanic White.” Sex was dichotomous with males coded as “1” and females coded as “2.” Smoking was also treated as a factor, comparing those that smoked “Some days” or “Not at all” to the referent group “Every day.”

REFERENCES

1. Entzel, P., et al. “Add Health Wave IV Documentation Report Cardiovascular and Anthropometric Measures.” (2009). Available at www.cpc.unc.edu.

2. Whitsel, E.A., et al. “Add Health Wave IV Documentation Report Measures of Glucose Homeostasis.” (2012). Available at www.cpc.unc.edu.

3. American Diabetes Association. "Diagnosis and Classification of Diabetes Mellitus." *Diabetes care* 34 Suppl 1 (2011): S62-9.

4. Whitsel, E.A., et al. “Add Health Wave IV Documentation Report Lipids.” (2013). Available at www.cpc.unc.edu.
